# Cu Nanowires and Nanoporous Ag Matrix Fabricated through Directional Solidification and Selective Dissolution of Ag–Cu Eutectic Alloys

**DOI:** 10.3390/ma15228189

**Published:** 2022-11-18

**Authors:** Jiaxing Xu, Jianjun Gao, Hongling Qin, Zhiyang Liu, Linpeng Zhu, Haibin Geng, Ligang Yao, Zhilong Zhao

**Affiliations:** 1 School of Mechanical Engineering and Automation, Fuzhou University, Fuzhou 350116, China; 2 State Key Laboratory of Advanced Welding and Joining, Harbin Institute of Technology, Harbin 150001, China; 3 State Key Laboratory of Solidification Technology, Northwestern Polytechnical University, Xi’an 710072, China

**Keywords:** Ag–Cu eutectic alloys, Cu nanowires, nanoporous Ag matrix, directional solidification, selective dissolution

## Abstract

Cu nanowires and a nanoporous Ag matrix were fabricated through directional solidification and selective dissolution of Ag–Cu eutectic alloys. Ag-39.9at.%Cu eutectic alloys were directionally solidified at growth rates of 14, 25, and 34 μm/s at a temperature gradient of 10 K/cm. The Cu phase in the Ag matrix gradually changed from lamellar to fibrous with an increase in the growth rate. The Ag matrix phase was selectively dissolved, and Cu nanowires of 300–600 nm in diameter and tens of microns in length were prepared in 0.1 M borate buffer with a pH of 9.18 at a constant potential of 0.7 V (vs. SCE). The nanoporous Ag matrix was fabricated through selective dissolution of Cu fiber phase in 0.1 M acetate buffer with a pH of 6.0 at a constant potential of 0.5 V (vs. SCE). The diameter of Ag pores decreased with increasing growth rate. The diameter and depth of Ag pores increased when corrosion time was extended. The depth of the pores was 30 μm after 12 h.

## 1. Introduction

Micro- and nanostructural materials with unique catalytic, adsorption, optical, thermal, electrical, and magnetic properties have been broadly used in biology, medicine, environment, energy, and other fields [1,2,3,4]. For nanostructured materials, more studies have focused on metal nanowires, nanorods, and quantum dots, especially metal nanowires. Metal nanowires are widely used as key materials for preparing microsensors, field emission electrodes, and magnetic memory systems due to their excellent electrical and mechanical properties [5,6,7]. Nanoporous metals have been applied in catalysts, sensors, fuel cells, micro-flow control, and other fields [8,9,10]. In recent years, nanopore manufacturing technology has been used for protein structure analysis, and it is expected to be integrated into various micro/nano devices by controlling the diameter and length of nanopore arrays and adjusting their surface properties [11,12].

Traditional preparation methods of metal nanowires mainly include the template-assisted method [13], physical vapor deposition [14], and the chemical liquid-phase method [15]. The template-assisted method is the most widely used in these preparation methods, and the structure of nanowires depends on the diameter and length of the template. Nanowire structure is obtained on a rigid template with 1D configuration by vapor deposition or electrochemical deposition. This method has high preparation efficiency, but the template is difficult to separate; this condition may damage the nanowires. In addition, the template application scope is small. The vapor deposition method can be divided into physical and chemical deposition methods. The reaction mechanism of the chemical liquid-phase method is to reduce metal ions to metal atoms by reducing agents and to promote the directional growth of copper nanocrystal nuclei by wrapping agents. Common methods for preparing nanoporous metals include dealloying and template methods [16,17]. The dealloying method generally has two methods: free corrosion and electrochemical corrosion. The components with more active electrochemical properties are dissolved by the corrosion solution due to the large difference in electrode potential between alloy components, and the remaining elements with stable electrochemical properties are reserved. Various nanoporous metals, such as gold, palladium, platinum, silver, and copper, have been successfully prepared by dealloying [18]. Recently, two advanced material processing methods, namely liquid metal-assisted dealloying [19] and vapor dealloying [20], have been discovered. Researchers have successfully prepared nanoporous structures such as FeCo and CaSi_2_ through these methods. The template method is adopted to prepare nanoporous metals by using materials with nanoporous structure. Common porous metals include AAO template, polycarbonate (PC) template, and hydrogen bubble template [21]. Another method for the preparation of porous metals by the Gasar method is also available. A porous structure is formed inside the metal through the direct directional solidification process, and the metal or alloy melt of gas (H_2_, N_2_, and O_2_) is dissolved by directional solidification saturation. The difference in the solubility of gas between the solid phase and the liquid phase is used, and the gas phase is precipitated in the solidification process and grows in coordination with the solid phase to prepare and arrange the porous metal with cylindrical pores in a directional manner [22]. Porous metals have excellent mechanical properties, chemical stability, and electrical and thermal properties. These metals have been used as templates for rare metal nanodot deposition or metal nanowire fabrication [23].

Ag–Cu alloy with two-phase fine wire structure is widely used in high-strength and high-conductivity materials due to its excellent mechanical properties and electrical conductivity after large deformations during cold drawing [24]. The research on Ag–Cu alloy mainly focuses on the influence of its microstructure and alloy composition on its properties, whereas the research on the preparation of micro- and nanostructures by Ag–Cu alloy is rare [25,26]. The selective phase dissolution technique was initially proposed by Hassel et al. Nanowire arrays and nanopore arrays were prepared by the combined directional solidification of eutectic alloy with selective dissolution [27,28]. Compared with the traditional preparation methods of nanowires and metal porous arrays, this technique does not require any template, and the obtained nanowires have a single-crystal structure with a high aspect ratio and have excellent mechanical and electrical properties [29]. As a self-organization process of surface diffusion, the dealloying method is difficult to use in controlling the morphology and size of micro/nano structures. However, the regulation of selective phase dissolution on the morphology of micro- and nanostructures occurs in the formation process of prefabricated alloys [30]. Therefore, a regular structure of directionally solidified eutectic alloys with different morphologies can be formed in the alloy by changing the growth rate [31,32]. At present, this technique has been applied to NiAl-X (Mo, W, Re, and Cr) and Ag–Cu alloy systems to prepare various single-crystal nanowires and metal nanopore arrays [33,34,35]. Although the directional eutectic Ag–Cu alloy has been prepared, the influence of growth rate on the microstructure and morphology of the alloy has not been deeply explored. Moreover, metallic nanowires and nanopore arrays prepared by this method can be used as working electrodes in micro-pH meters [22] and as excellent templates for electrochemical deposition of micro/nano structures [36].

**Figure 1:**
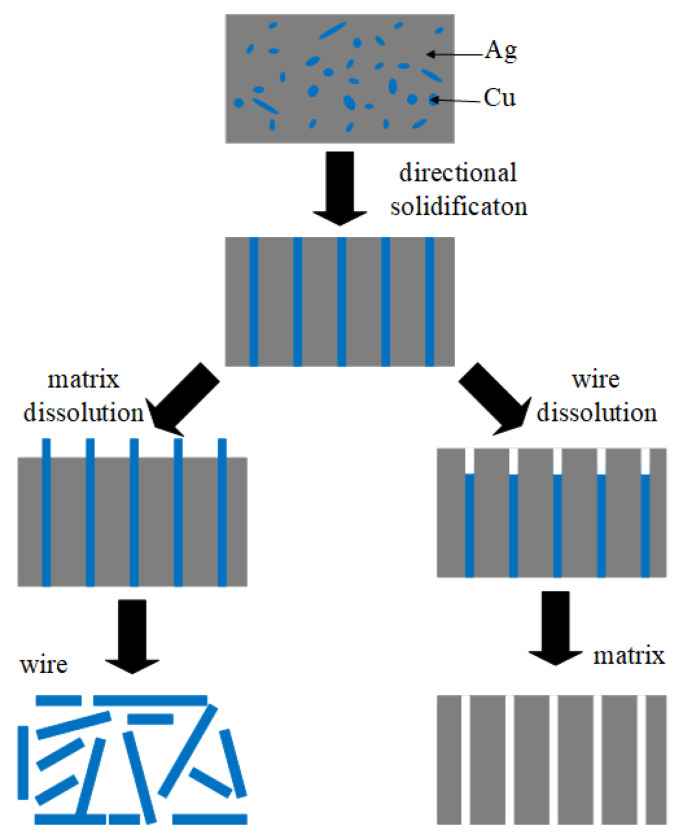
Roadmap for the preparation of Cu nanowires and nanoporous Ag matrix.

In this study, Cu nanowires and Ag nanopore templates were prepared by a selective phase dissolution of directionally solidified Ag–Cu eutectic alloy. The effects of different growth rates on the microstructure of Ag–Cu eutectic alloy and of the dissolution duration on the morphology of Cu nanowires and a nanoporous Ag matrix fabricated through selective phase dissolution were studied. The electrochemical reaction mechanism in the preparation of two types of micro/nano structures was also analyzed, thereby providing a reference for optimizing the preparation of Ag–Cu eutectic alloy micro/nano structures by selective phase dissolution. The roadmap in Figure 1 shows the processing of the corresponding directionally solidified Ag–Cu system, which involves the preparation process of Cu nanowires and Ag pores. When the Ag matrix was selectively dissolved and the Cu nanowires were processed, the Ag pores were prepared by a dissolution of Cu fiber phase.

## 2. Materials and Methods

Pre-alloys were prepared by mixing Ag particles (99.99 wt.%) and Cu particles (99.99 wt.%) in the ratio of Ag-39.9at.%Cu. A Ag–Cu eutectic ingot with uniform composition was obtained by repeated melting in an electromagnetic induction melting furnace. The cylindrical pre-alloy castings were machined as a series of test rods with a length of 100 mm and diameter of Φ4 mm by using wire-electrode cutting. The test rod was polished until the surface had no oxide layer and cleaned by ethanol in an ultrasonic cleaner. Then, it was loaded into a corundum tube and solidified by Bridgman induction heating. Directional solidification experiments were conducted at a temperature of 870 °C. The temperature gradient was 10 K/cm, and the growth rates were 14, 25, and 34 μm/s.

All electrochemical experiments were carried out on a CS150H electrochemical workstation. A conventional three-compartment cell was employed in the electrochemical tests, where a Pt foil with a surface area of 2 cm^2^ and a saturated calomel electrode (SCE) were the counter and reference electrodes, respectively. The working electrodes included the 2 mm thick cylindrical specimen, whose back was welded with copper wires and embedded in the epoxy resin with the working surface exposed to the solution. Because the buffer solutions had a stable pH value, the solutions used for electrochemical experiments were 0.1 M borate buffer (pH = 9.18) and 1 M acetate buffer (pH = 6.0). The scanning range of the potentiodynamic polarization curve was 0 to 1.5 V_SCE_, and the scanning rate was 10 mV/s.

An MDS400 metallographic microscope and OptecOPTPRO metallographic image analysis software were used to observe the microstructures of nanowires and pores and determine the characteristic size of the phase. A Nova NanoSEM 230 High-resolution field emission scanning electron microscope was used to analyze the microstructure of Cu nanowires and Ag pores.

## 3. Results and Discussion

### 3.1. Microstructure of Ag–Cu Eutectic Alloy

**Figure 2:**
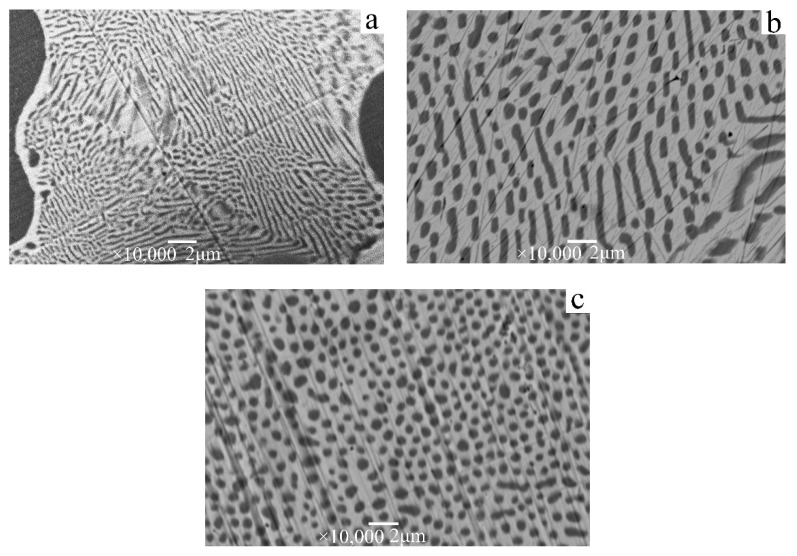
SEM photographs of cross section of Ag-39.9at.%Cu eutectic alloy under different growth rates: (**a**) 14, (**b**) 25, and (**c**) 34 μm/s.

The structure and morphologies of the directionally solidified Ag–Cu eutectic alloys with growth rates of 14, 25, and 34 μm/s are illustrated in Figure 2. The Ag matrix is silver–white, and the Cu phase is black. As shown in Figure 2, the Cu phase in the Ag matrix gradually changed from lamellar to fibrous as the growth rate increased. When the growth rate is 14 μm/s (Figure 2a), the Cu phase shows a completely lamellar structure. When the growth rate is 25 μm/s (Figure 2b), the Cu phase reveals a mixed fibrous and lamellar microstructure. When the growth rate increases to 34 μm/s (Figure 2c), the Cu fibrous phase is uniformly distributed in the sliver matrix. With an increase in the growth rate, the diameter of the Cu fiber phase decreases, and the spacing decreases.

According to the directionally solidified nucleation theory of eutectic alloys, the ratio of two-phase volume fractions of binary eutectic alloys is approximately the critical value of π^−1^ ≈ 0.318. Lamellar or rod-like eutectic alloys can be formed in the eutectic. A lamellar eutectic alloy is formed when the volume fraction of the second phase is greater than 31.8%. When the volume fraction of the second phase is less than 31.8%, a rod eutectic alloy is formed. The selected volume fraction of the Ag–39.9at.%Cu system is the only one that matches exactly the theoretical value of π^−1^. At this time, the volume fraction of copper phase is 31.8%. According to the layer–rod transition principle proposed by Jackson Hunt [37], when the interface energy of the rod-like morphology is lower than that of the lamellar structure, the lamellar structure begins to transform into the rod-like structure. Therefore, the selection of suitable directional solidification parameters is an important condition for obtaining fibrous eutectic. The relationship between the growth rate v and the eutectic spacing *λ* at a certain temperature gradient is as follows:

(1) $$C_{1}=\lambda^{2}v$$

The relationship between the fiber size a and the growth rate v is obtained as follows:

(2) $$C_{2}=a^{2}v$$

**Figure 3:**
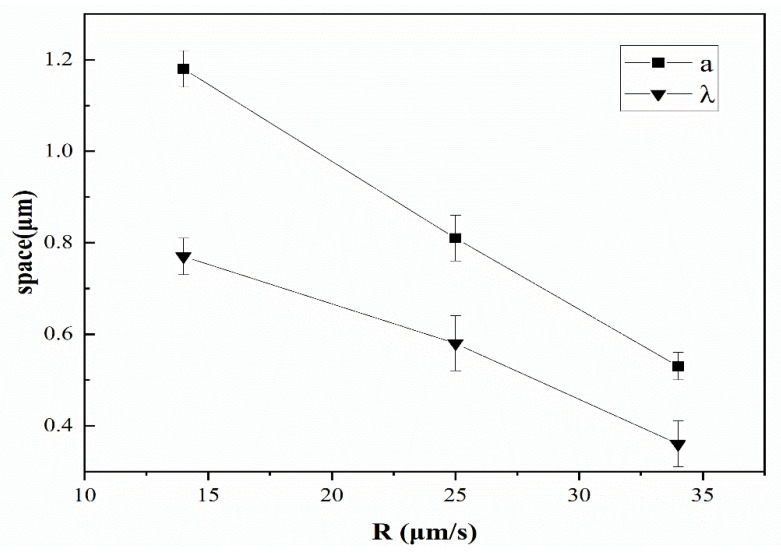
Variation in fiber size (a) and fiber spacing (λ) with growth rate v.

Because *C*_1_ and *C*_2_ in Equations (1) and (2) are constants, the fiber size a and eutectic spacing λ can be controlled by changing the growth rate v. As shown in Figure 3, when the growth rate increases from 14 μm/s to 34 μm/s, fiber size and fiber spacing decrease from 1180 and 770 nm to 360 and 530 nm. According to the component supercooling theory [37], undercooling of alloy during directional solidification should not be extensive in order to obtain a regular fibrous eutectic. At a certain temperature gradient, higher growth rate and a greater amount of corresponding undercooling indicate smaller fiber size.

### 3.2. Preparation of Cu Nanowires by Selective Dissolution of Ag Matrix

**Figure 4:**
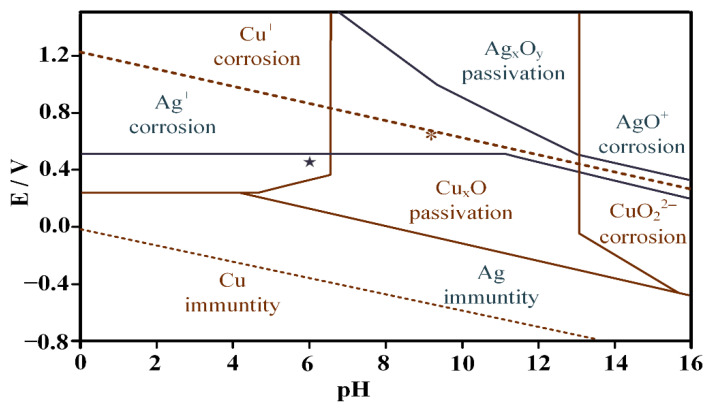
Combined Pourbaix diagram for Ag and Cu. The gray asterisk (★) indicates the electrochemical conditions used to prepare nanoporous Ag matrix. The red asterisk (*) indicates the conditions used to prepare Cu nanowires [34].

**Figure 5:**
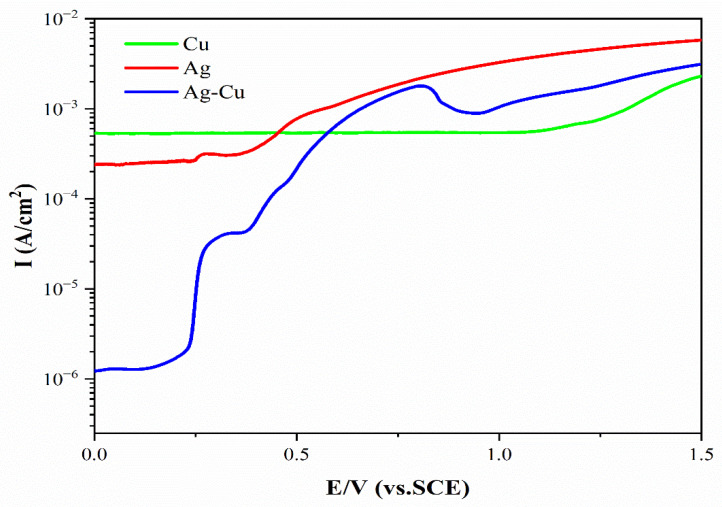
Potentiodynamic polarization curves (vs. SCE) of pure Ag, Cu, and Ag–Cu eutectic in the 0.1 M borate buffer solutions at a scan rate of 10 mV/s.

An appropriate potential and pH value are required for the selective dissolution of the Ag matrix to obtain Cu nanowires. The combined Pourbaix diagram of pure Ag and Cu is shown in Figure 4, where corrosion-free zones, corrosion zones, and passivation zones for each element are divided. Cu elements should be in passivation or corrosion-free zones to prepare Cu nanowires, whereas Ag elements should be in corrosion zones. When the corrosion potential is greater than 0.58 V and the pH value is less than 11, Ag is corroded to form Ag^+^ or oxidized toform AgO. The potentiodynamic polarization curve of pure Ag, Cu, and Ag–Cu eutectic alloys is shown in Figure 5. When the corrosion potential is lower than 1.2 V, with an increase in the corrosion potential, the current density of pure Cu is almost unchanged. When the corrosion potential is higher than 1.2 V, the corrosion current density increases sharply. The current density of pure Ag remains almost unchanged when the corrosion potential is less than 0.4 V, and the current density increases with an increase in corrosion potential when the corrosion potential is higher than 0.4 V. The potentiodynamic polarization curve of Ag–Cu eutectic shows that when the potential is less than 0.7 V, the corrosion current density increases with an increase in corrosion potential. When the corrosion potential is between 0.7 and 1.0 V, the current density decreases with an increase in corrosion potential, probably due to the continuous oxidation of Ag in the alloy and the continuous passivation of Cu. The current density increases when the potential increases. Therefore, 0.1 M borate buffer with a pH of 9.18 and a 0.7 V (vs. SCE) constant potential were selected for selective dissolution of the Ag matrix to prepare Cu nanowires.

**Figure 6:**
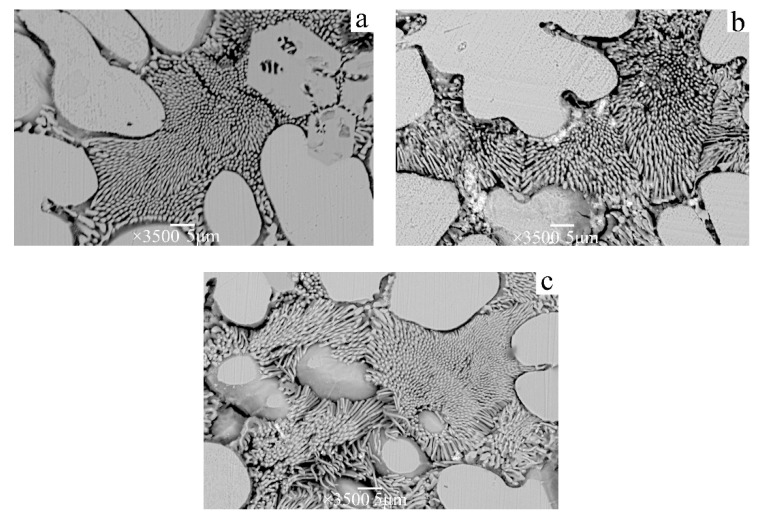
SEM images of Cu nanowires in different dissolution time periods with a growth rate of 34 μm/s: (**a**) 4 h, (**b**) 12 h, and (**c**) 24 h.

**Figure 7:**
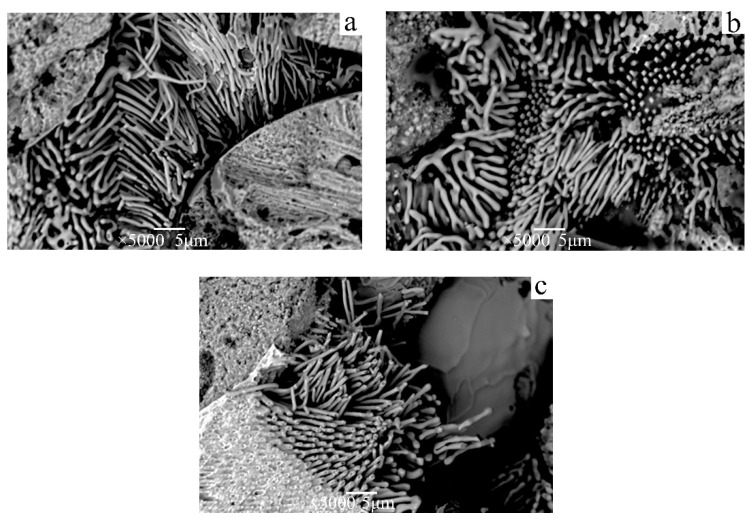
Microstructure of Cu phase at different directional solidification rates: (**a**) 14, (**b**) 25, and (**c**) 34 μm/s.

The Ag matrix phase is selectively dissolved at a constant potential of 0.7 V (vs. SCE). In the early stage of corrosion, silver oxide precipitates on the surface of the electrode, and the Ag matrix dissolves rapidly. As the reaction proceeds, a yellow layer of Cu_2_O is formed on the surface of the Cu, and some Cu_2_O solution is oxidized to black copper oxide and copper hydroxide. This double-layer passivation film structure effectively prevents the dissolution of Cu nanowires. The microstructure and morphology of Cu nanowires prepared in different corrosion times are illustrated in Figure 6. In the early stage of corrosion, the Ag matrix near the copper nanowires is rapidly dissolved, and the silver–white AgOH flocculent precipitate is exposed on the surface of the copper nanowires (Figure 6b). After corrosion for 24 h, the dendritic Ag matrix away from the Cu nanowires dissolves gradually, and the length of Cu nanowires increases continuously. The longest Cu nanowires can reach more than 20 microns and the diameter is approximately 200–400 nm. The microstructure of Cu nanowires grown at different growth rates is illustrated in Figure 7. Cu presents a thin banded structure at the growth rate of 14 μm/s (Figure 7a). At the growth rate of 25 μm/s (Figure 7b), Cu is a fibrous and lamellar mixed structure with a large amount of lamellar Cu bifurcating. When the growth rate further increases to 34 μm/s (Figure 7c), the distribution of Cu nanowires with completely fibrous structure is formed.

In the early stage of corrosion, copper is converted into hydration ions in the form of low valence, and Cu_2_O is formed on the surface of copper:

(3) $$2\mathrm{Cu}+2{\mathrm{OH}}^{-}\to {\mathrm{Cu}}_{2}O+2e^{-}$$

Subsequently, Cu is in the activation–passivation transition zone, and the metal surface generates bivalent or trivalent transition oxides. The oxide structure is unstable and is accompanied by the dissolution and re-generation of the oxide film, as follows:

(4) $${\mathrm{Cu}}_{2}O+2{\mathrm{OH}}^{-}\to 2\mathrm{CuO}+H_{2}O+2e^{-}$$

In the stable passivation stage of copper, a composite passivation film with good corrosion resistance is formed on the surface, which is a mixed structure of Cu_2_O, CuO, and Cu(OH)_2_.

(5) $$2\mathrm{CuO}+2{\mathrm{OH}}^{-}\to 2\mathrm{Cu}{\left(\mathrm{OH}\right)}_{2}+2e^{-}$$

The reaction process of dissolving Ag substrate into Ag ^+^ ions is as follows:

(6) $$2\mathrm{Ag}+2H^{+}\to 2{\mathrm{Ag}}^{+}+H_{2}$$

With the dissolution reaction of Ag, white flocculent precipitate AgOH is formed, as follows:

(7) $${\mathrm{Ag}}^{+}+{\mathrm{OH}}^{-}\to \mathrm{AgOH}$$

**Figure 8:**
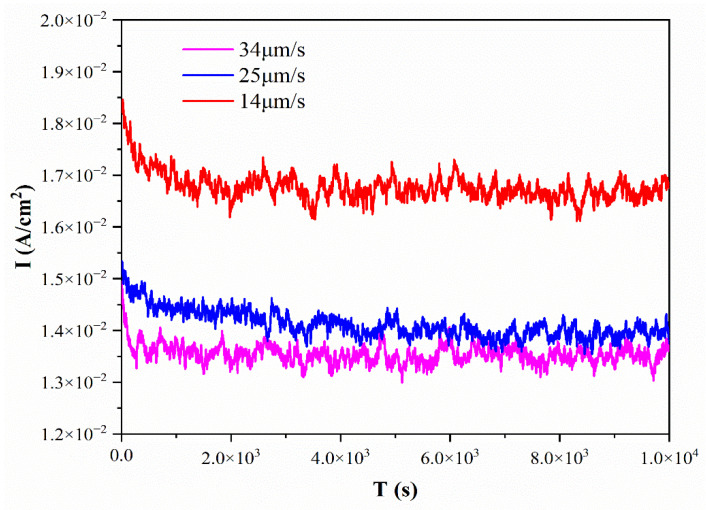
Current density curve with time extension of Ag–Cu eutectic alloy in 0.1 M borate buffer at different growth rates.

The current density curve with the time extension of selective corrosion of Ag–Cu eutectic alloy with different growth rates is shown in Figure 8. As shown in Figure 8, due to the passivation of the alloy surface during the corrosion process, the current density decreases rapidly at the initial stage of corrosion. With the formation and dissolution of the passivation film, the current density gradually remains stable. The corrosion current density of Ag–Cu eutectic alloy grown at 14 μm/s is the lowest, and the current density fluctuates around 1.35 × 10^−2^ A/cm^−2^. The corrosion current density of 25 μm/s Ag–Cu is slightly higher than that of 14 μm/s Ag–Cu, and the current density is approximately 1.40 × 10^−2^ A/cm^−2^. The corrosion current density is the highest when the growth rate is 34 μm/s, and the current density fluctuates at approximately 1.65 × 10^−2^ A/cm^−2^. The reason for the sharp fluctuation in the current is that in the process of removing the Ag matrix, the corrosion and dissolution of the Ag matrix and the destruction of the Cu passivation film constitute a dynamic balance. Under the same corrosion time, the corrosion efficiency of Ag–Cu eutectic alloy grown at 34 μm/s is the highest, and the length of Cu nanowires is the longest (Figure 7c), whereas the corrosion efficiency of eutectic alloy grown at 14 μm/s is the lowest. With an increase in growth rate, the spacing of copper nanowires and the diameter of copper fibers decrease, and copper presents a complete nanowire structure. The corrosion rate of Ag–Cu eutectic alloy with high growth rate is efficient under the same corrosion time.

### 3.3. Preparation of Nanoporous Ag Matrix by Selective Dissolving of Cu Fiber Phase

**Figure 9:**
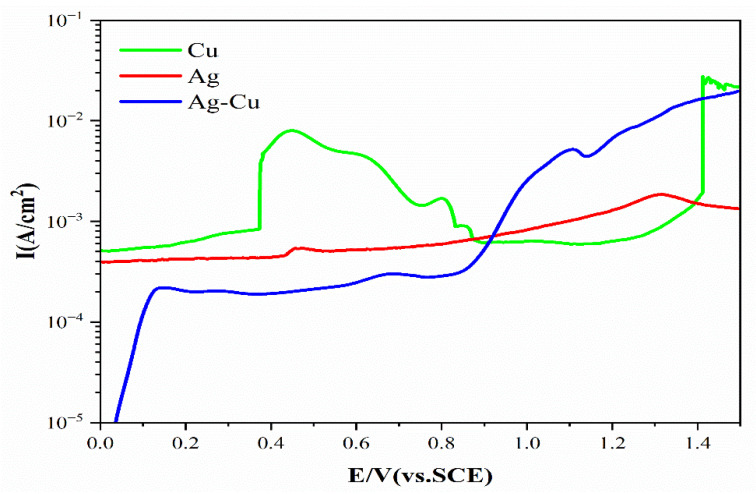
Potentiodynamic polarization curves (vs. SCE) of pure Ag, Cu, and Ag–Cu eutectic in the 1.0 M acetate buffer solutions at a scan rate of 10 mV/s.

The potentiodynamic polarization curve of pure Ag, Cu, and Ag–Cu eutectic alloys in 1.0 M acetate buffer is shown in Figure 9. As shown in Figure 9, when the corrosion potential increases from 0 V to 0.5 V, the current density of Cu increases with the increase in corrosion potential, and the current density reaches a peak of 0.0255 A/cm^2^. The current density decreases with the increase in corrosion potential when the potential is between 0.5 and 1.0 V. The Cu is in an over-passive state and the passive film breaks when the potential increases by 1.0 V. The current density increases sharply with the increase in potential. The current density of Ag increases with the increase in corrosion potential, and it has peaks at 0.5 and 1.3 V. The current density of Ag–Cu eutectic alloy increases with the increase in corrosion potential. When the corrosion potential is lower than 0.15 V, the current density increases sharply with the increase in potential. When the corrosion potential is between 0.15 and 0.8 V, the current density tends to be stable, mainly due to the combined effect of Cu passivation and Ag corrosion. When the potential exceeds 0.8 V, the current density increases with the increase in corrosion potential. Therefore, to obtain Ag porous template for selective corrosion removal of Cu, the corrosion potential should be selected at 0.5 V. At a potential of 0.5 V, black Cu phase is corroded, whereas silver–white Ag phase is oxidized and free of corrosion.

The corrosion solution for preparing Ag porous template by selective dissolution of Cu is 1.0 M acetate buffer solution with a pH of 6, and the corrosion potential is 0.5 V. The oxide of Cu has effective solubility in acetate buffer solution. Therefore, no oxide layer is formed during the dissolution process. At the same time, sky-blue solution and copper ions are generated. The specific reaction equation is as follows:

(8) $$2\mathrm{Cu}+2H^{+}\to {\mathrm{Cu}}^{2+}+H_{2}$$

**Figure 10:**
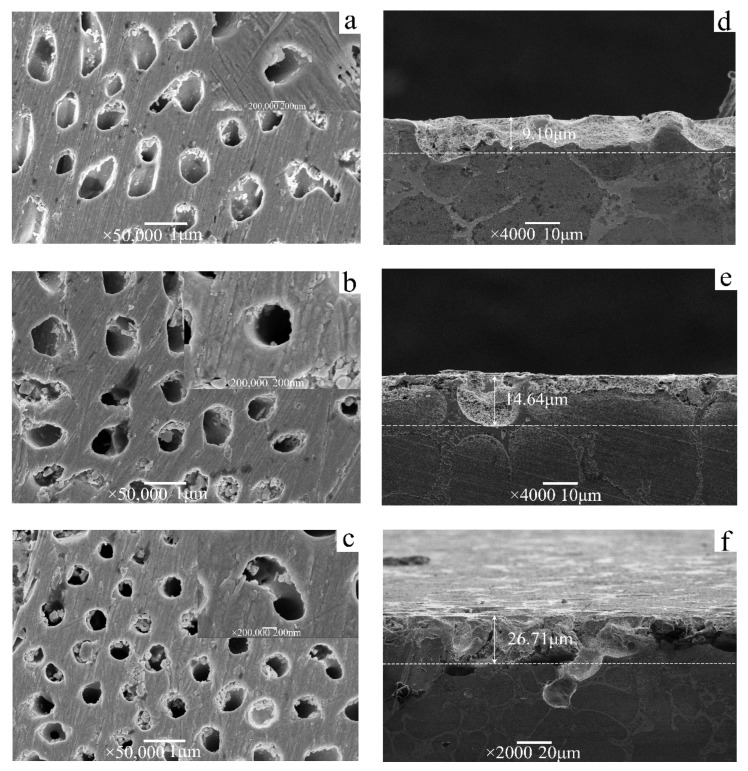
The transverse and longitudinal sections of the nanoporous Ag matrix prepared by a constant potential of 0.5 V with a growth rate of 34 μm/s in different dissolution durations: (**a**,**d**) 2 h, (**b**,**e**) 4 h, and (**c**,**f**) 8 h.

The nanoporous Ag matrix obtained under different dissolution durations at a constant potential of 0.5 V the illustrated in Figure 10. When the dissolution duration is 2 h (Figure 10a), shallow holes are exposed on the surface of the Ag substrate, and the diameter of the holes ranges from 200 nm to 600 nm. The diameter of the holes is nearly that of Cu nanowires (Figure 7). A small number of corrosion particles remain at the edges of the holes, and the outline of the sidewall in the holes is clearly visible. When the dissolution duration increases to 4 h (Figure 10b), the Cu nanowires inside the Ag hole are corroded and removed, and the edge structure of the hole is clearly visible. When the dissolution duration is 8 h (Figure 10c), the corrosion depth of the hole further increases, and most holes on the surface of the Ag matrix are exposed. With the extension in dissolution duration, some gaps are found at the edge of the hole. The Cu fiber phase is continuously dissolved, and the hole depth of the Ag porous template increases (Table 1). Therefore, the Ag porous structures with different depths can be prepared by controlling the dissolution duration.

**Figure 11:**
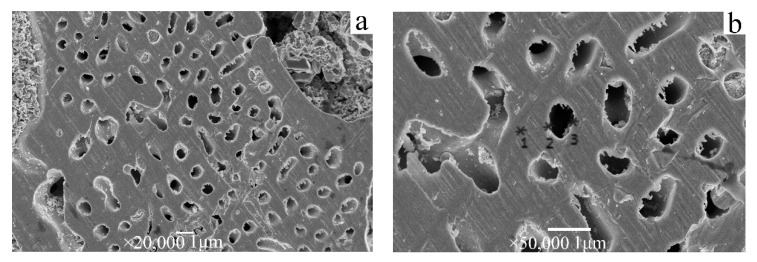
Porous Ag morphology at a growth rate of 34 μm/s with a dissolving time of 2 h. (**a**) A high-magnification photo of a pore in (**b**) shows the three points of ∗1, ∗2, and ∗3 for analysis of energy dispersive spectra.

Table 2 shows the energy dispersive spectra (EDS) analysis results of the nanoporous Ag matrix in Figure 2 at different positions. As shown in Table 2, Ag and Cu elements are present on the surface of the matrix (position *1 in Figure 11) and near the edge of the pores (position *2 in Figure 11) as well as in the edge of the pores (position *3 in Figure 11). The EDS analysis results in Table 2 show that from the surface of the porous Ag matrix to the edge of the hole, the content of Cu decreases and the content of Ag increases. The kinetic polarization curve in Figure 9 shows that Cu is in the state of corrosion, whereas Ag is in the oxidized state at 0.5 V constant potential. Therefore, at a constant potential of 0.5 V, the Cu elements are continuously removed by corrosion, whereas the Ag elements are passivated and are free from corrosion. Cu nanowires are removed by selective dissolution, and the Ag substrate edge is partially corroded with an extended dissolution time (Figure 10).

## 4. Conclusions

Cu nanowires were obtained by selectively dissolving the Ag matrix, and a porous Ag structure was obtained by selectively dissolving the Cu nanowires. The conclusions are as follows:

(1) With the increase in growth rate, the diameter and spacing of the Cu fiber phase decreased. When the growth rate was 14 μm/s, the Cu phase showed a long lamellar structure. With the increase in the growth rate, the Cu phase gradually changed from lamellar to fibrous. When the growth rate was 34 μm/s, the Cu phase became completely fibrous;
(2) Cu nanowires were obtained by selective dissolution of the Ag matrix in 0.1 M borate buffer with a pH of 9.18 at a constant potential of 0.7 V_SCE_. With the extension in dissolving duration, the length of Cu nanowires increased. The length of Cu nanowires could reach more than 20 microns in 12 h, and the diameter was 200–600 nm;
(3) A nanoporous Ag matrix was obtained by selective dissolution of the Cu fiber phase in 1 M acetate buffer with a pH of 6.0 at constant potential of 0.5 V_SCE_. With the increase in dissolving duration, the depth of the pores increased, whereas the diameter of the holes remained almost unchanged. When the dissolving duration was 12 h, the depth of the pores could reach 30 μm.

## Figures and Tables

**Table 1:** Diameter and depth of Ag holes at different dissolution times.

Dissolution Duration	Pore Diameter (nm)	Dissolving Depth (μm)
2 h	4 h	8 h
490.9	549.5	751.7
9.10	14.64	26.71

**Table 2:** Energy dispersive spectroscopic results of three points of *1, *2, and *3 in Figure 11.

Test Points	Element	Mass Fraction (%)
*1	Ag	83.76
	Cu	16.24
	O	/
*2	Ag	81.33
	Cu	9.85
	O	8.82
*3	Ag	78.38
	Cu	9.26
	O	12.36

## Data Availability

The data that support the findings of this study are available from the corresponding author upon reasonable request.
